# The effects of an 8-week Exer-Genie-assisted speed and ABC drill training program on speed, hamstring strength, and vertical jump performance in female football players

**DOI:** 10.1186/s13102-026-01744-3

**Published:** 2026-05-13

**Authors:** Yusufcan Keskin, Mehmet Yavuz Taşkıran, Gürkan Elçi

**Affiliations:** 1 Department of Physical Education and Sports, İstanbul Gedik University, İstanbul, Türkiye; 2 Faculty of Sports Science, İstanbul Rumeli University, İstanbul, Türkiye; 3 Faculty of Sports Science Department of Recreation, Bartın University, Bartın , Türkiye

**Keywords:** Resistance Training, Athletic Performance, Football

## Abstract

**Purpose:**

The increasing physical demands of modern women’s football necessitate integrated training approaches capable of simultaneously enhancing acceleration, force production, and neuromuscular control. Although devices such as the Exer-Genie® are frequently implemented in resisted sprint training, scientific evidence regarding their effectiveness in adolescent female athletes remains limited. Therefore, this study aimed to examine the effects of an eight-week Exer-Genie®-assisted speed and agility–balance–coordination (ABC drill) training program on sprint performance, hamstring muscle strength, and vertical jump performance in young female football players.

**Methods:**

A total of 37 licensed female football players competing in the Turkish Women’s Second League (mean age = 14.4 ± 1.4 years) voluntarily participated in the study. Participants were randomly assigned to an experimental group (*n* = 20) or a control group (*n* = 17). The experimental group completed an Exer-Genie®-assisted sprint and agility–balance–coordination training program twice per week for eight weeks, while the control group continued their regular training. Pre- and post-intervention assessments included 0–10 m, 10–30 m, and 30 m sprint performance, hamstring muscle strength and impulse values, and countermovement jump performance.Data were analyzed using repeated-measures ANOVA.

**Results:**

Significant time × group interactions were observed for 0–10 m sprint time (3.9% improvement; F = 10.17, *p* = .003, η² = 0.038) and 30 m sprint time (3.8% improvement; F = 21.08, *p* < .001, η² = 0.020). Hamstring maximal strength increased by approximately 20% (left: F = 15.94, *p* < .001, η² = 0.03), while hamstring impulse improved by approximately 36% (left: F = 10.62, *p* = .002, η² = 0.05; right: F = 9.75, *p* = .004, η² = 0.03). No significant improvements were observed in lower-limb strength asymmetry.

**Conclusion:**

Eight weeks of Exer-Genie^®^-assisted speed and ABC drill training significantly enhanced acceleration and hamstring strength parameters in young female football players. The observed improvements (~ 4% in sprint performance, ~ 20% in maximal strength, and ~ 36% in impulse capacity) support the potential usefulness of combining resisted sprint training with neuromuscular coordination drills.

**Trial registration:**

ClinicalTrials.gov, NCT07274566.Registered 28.11.2025 Retrospectively registered.

**Supplementary Information:**

The online version contains supplementary material available at 10.1186/s13102-026-01744-3.

## Introduction

The recent increase in interest in women’s football has not only driven greater financial investment but has also contributed to the development of a more professional structure for teams, leagues, and the overall organization of the sport [1, 2]. This transformation has considerably elevated the athletic performance demands in modern women’s football [3].

Recent systematic review and meta-analysis evidence has further highlighted that sprint performance, lower-limb power, and change-of-direction ability are key determinants of success in women’s football. In particular, Compton et al. demonstrated that these physical qualities clearly differentiate performance levels in female players, emphasizing the importance of targeted neuromuscular training interventions. Additionally, meta-analytic findings from plyometric and strength-based training studies in female soccer players have shown significant improvements in sprint and vertical jump performance, supporting the implementation of structured speed- and power-oriented training programs in this population [4–6].

Specifically, time-motion analyses in female soccer indicate that players typically cover approximately 9–11 km per match, including 20–40 short sprint efforts predominantly occurring over distances shorter than 20 m [7]. These high-intensity actions are most frequently observed during critical phases such as offensive and defensive transitions, highlighting the importance of acceleration and short-distance sprint performance in match success.Particularly in talent identification processes conducted in younger age groups, these performance requirements have become an important determining criterion [8, 9]. Short-distance sprint performance, especially acceleration between 5 and 20 m, is vital in this context as the majority of sprints performed during a match occur over short distances rather than at absolute maximum velocity [10].

Sudden accelerations, decelerations, and changes of direction performed during match play are among the primary determinants of performance [11]. These capabilities are closely linked to neuromuscular characteristics such as lower-limb strength and power [12].The change-of-direction ability of female football players is closely associated with eccentric muscle strength. Female soccer players with higher eccentric strength are able to better control velocity during sudden decelerations performed at maximal speed, enabling faster directional changes [13].Additionally, sprint performance plays a key role in football, as high-speed running and sprint actions are important components of match performance [14]. Resisted sprint training (RST) includes various methods such as weighted sleds, parachutes, weighted vests, and motorized resistance systems (e.g., Exer-Genie and 1080 Sprint), all of which aim to increase horizontal force production during the acceleration phase [15–19].Recent meta-analytic findings confirm that RST provides significant improvements in 10 m acceleration performance, with some evidence also suggesting positive effects on vertical jump height [20, 21]. This method increases horizontal force-production capacity by creating a resistive force acting opposite to the direction of the athlete’s center-of-mass movement, requiring greater force application during the acceleration phase.

As a result, athletes are forced to produce higher propulsive forces and improve neuromuscular coordination, particularly in the early stages of sprinting [22, 23].

In recent years, portable devices capable of providing adjustable resistance have been developed. The Exer-Genie^®^ (Thousand Oaks, CA) device offers horizontal resistance through a flexible rope and rotating spool mechanism. While longitudinal performance data for the Exer-Genie are more limited compared to motorized or sled systems, recent studies have quantified the horizontal resistance across its different settings, supporting its use as a controlled field-based tool for producing systematic resistance. Studies have demonstrated a strong relationship between load settings and the horizontal force produced by the device, with higher force outputs observed in 60 m models compared to 36 m models at equivalent load settings. Additionally, discrepancies between the indicated load level and the actual force output have been reported, highlighting an important consideration when prescribing training loads [15].Furthermore, evidence from motorized resistance systems (e.g., 1080 Sprint) suggests that light-to-moderate loads (≤ 10–15% body mass) can provide an effective speed-strength stimulus while minimizing the risk of disrupting sprint technique [17]. Sled-integrated RST programs have also demonstrated the ability to simultaneously improve sprint performance, vertical jump height, and posterior chain mechanical functions in both youth and professional football players [18, 24].

Alongside improvements in performance, injury risks have also increased. While hamstring injuries were historically classified among the most common injuries in elite male football [25, 26], they are now also frequently observed among elite female players [27, 28]. Epidemiological data suggest that hamstring injuries are among the most common time-loss injuries in elite female soccer, highlighting the necessity of force-based and sprint-oriented training strategies [29]. A UEFA report indicated that hamstring injuries account for approximately 12–24% of all injuries, with teams typically experiencing around 4–6 hamstring injuries per season [30]. Previous studies have emphasized that insufficient exposure to high-speed actions increases the risk of hamstring injury, highlighting the necessity of incorporating specific speed and force-oriented exercises into training programs [31, 32].The NordBord device, commonly used to evaluate hamstring strength, provides high precision in eccentric and isometric force assessments [33]. Assessing eccentric hamstring strength alongside sprint performance is critical, as evidence suggests that while heavy RST may enhance force production, its superiority over unresisted sprinting can vary depending on the intervention duration and athlete profile [19]. Likewise, vertical jump performance can be analyzed in detail using force platforms through the countermovement jump (CMJ) test. CMJ is not merely a generic measure but a reflection of lower-limb neuromuscular function relevant to sprint capacity; recent evidence shows significant associations between CMJ-derived variables and sprint performance in professional players [34]. Furthermore, CMJ serves as a practical marker of acute neuromuscular fatigue and demonstrates high reliability in elite youth football [35, 36].

“ABC drills” (A-Skip, B-Skip, C-Skip, etc.), applied for warm-up and fundamental motor skill development, are designed to teach sprint mechanics and improve running efficiency [37, 38]. Biomechanical evidence suggests that while these drills do not exactly replicate running mechanics, they target specific coordination and technical components [39]. Specifically, drills like the B-skip have shown meaningful associations with 5 m and 20 m sprint performance, making them a justified technical addition to sprint-based interventions [40]. In addition, resisted sprint training (RST) aims to enhance the capacity for horizontal force production and strengthen the initial acceleration phase. By combining technical drills with RST, coaches can target both the mechanical efficiency and the horizontal force-production capacity required for elite performance.

Despite the growing body of literature on resisted sprint training (RST), limited research has specifically examined the combined effects of Exer-Genie-assisted sprint training and ABC drills on sprint performance, hamstring strength, and neuromuscular characteristics in young female football players. Furthermore, the effectiveness of such integrated, field-based interventions in this population remains unclear.

Based on these considerations, the aim of this study is to investigate the effects of an 8-week training program combining Exer-Genie-assisted resisted sprints and ABC drills on speed, hamstring strength, and vertical jump performance in young female football players.

## Methods

### Participants

**Fig. 1:**
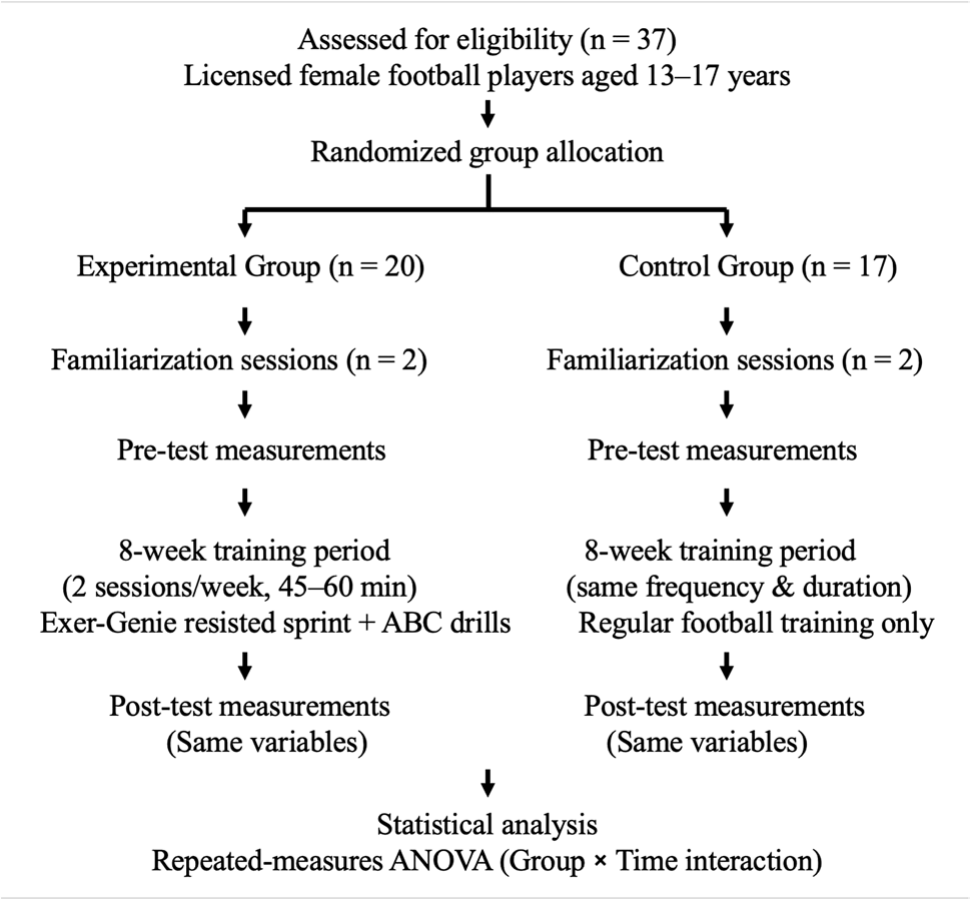
Experimental design of the study

The study included female football players from the Pendik Güven Women’s Football Team, with a mean age of 14.4 ± 1.4 years. Participants were licensed by the Turkish Football Federation, had been regularly engaged in football training for at least two years. As part of the standard licensing procedure at the beginning of the season, all players underwent comprehensive medical screening prior to participation in competitive sport. Additionally, none of the participants had a history of injury or any health condition that could adversely affect performance. (Fig. 1).

Prior to the commencement of the study, all players were familiarized with the testing protocols and training systems. During the first week, a familiarization (demo) session was conducted in which the procedures were explained in detail and participants were allowed to practice all tests and training applications to ensure proper understanding and execution.

The sample size to be used in the study was determined prior to data collection through a power analysis performed using the JASP software (v.0.95.4). The description of the power analysis was condensed to emphasize that statistical power was primarily dependent on the expected effect size. Based on the power curve, a sample size of approximately 20 participants per group would provide a statistical power of at least 69.3% (1 − β ≥ 0.693) for detecting large effect sizes (|δ| > 0.8). However, the probability of detecting smaller effect sizes (|δ| < 0.636) was considerably lower, indicating limited sensitivity for small to moderate effects.

Written informed consent was obtained from the parents or legal guardians, assent was obtained from the players themselves, and permission was granted by the club administration prior to the start of the study.

### Nordbord hamstring test protocol (ISO Prone)

The Nordic Hamstring Exercise (NHE) is a highly reliable method for assessing eccentric hamstring strength and has been validated through the NordBord system [41]. Its test–retest reliability is reported as ICC ≈ 0.99 [42]. Therefore, NHE is accepted as a standard reference method for hamstring strength assessment [43].

#### ISO Prone (0°)

This is an end-length isometric test performed with the knees in full extension (0° flexion). Participants begin in a kneeling position on the NordBord device, maintaining their legs straight (full extension), and apply maximal hamstring force by pulling their heels upward for 3–5 s.

Before testing, participants performed a standardized warm-up including submaximal contractions to ensure readiness and reduce injury risk [44].Participants completed three maximal isometric trials, each lasting approximately 5 s, with 30–60 s of rest between trials to minimize fatigue [45].All trials were visually monitored, and any trial with improper technique or compensatory movements (e.g., hip flexion or loss of position) was discarded and repeated.The highest peak force (N) obtained from the trials was used for statistical analysis, as commonly recommended in NordBord testing protocols.

Both limbs were assessed simultaneously, and force data were recorded in real time via the NordBord software system [46].

### 30 m sprint test (smartspeed timing gates)

The sprint test was conducted on a flat indoor surface while wearing standard sports shoes to eliminate the potential influence of wind. Sprint times were recorded using the SmartSpeed electronic timing system (Fusion Sport, Coopers Plains, Australia). Each athlete performed two maximal 30 m sprint trials, and the best time was used for statistical analysis. In addition, 0–10 m and 10–30 m split times were recorded.

All sprints were performed at maximal effort. Participants initiated the sprint from a standing start using a two-point stance, positioned behind the first timing gate to prevent early beam triggering. Electronic timing gates are considered a reliable method for measuring short sprint performance in the literature [47, 48].

### Countermovement Jump (CMJ) test

Vertical jump performance was assessed using the countermovement jump (CMJ) test. CMJ is widely recognized for its high validity in measuring explosive power and demonstrates strong reliability across repeated measurements in athletes [49]. The ForceDecks system used in this study has been shown in previous research to provide highly accurate load-cell force measurements and to be largely comparable to laboratory-grade equipment [50]. For this reason, CMJ and ForceDecks outputs were accepted as reliable indicators of lower-limb explosive performance.

The CMJ test was performed using portable dual force plates (ForceDecks, VALD Performance, Brisbane, Australia), with a sampling frequency of 1000 Hz. Participants performed the jumps with hands placed on the hips to eliminate arm swing, starting from an upright standing position followed by a rapid downward movement and an immediate vertical jump. Each participant completed three maximal CMJ trials, with approximately 30–60 s of rest between trials to minimize fatigue. All jumps were performed at maximal effort, and the highest value obtained from the trials was used for statistical analysis.

### Training protocol and use of the Exer-Genie

The participant group completed Exer-Genie^®^-assisted speed and ABC drill training twice per week for eight weeks (a total of 16 sessions). The control group continued their regular football training program throughout the study period, with a similar weekly training frequency and session duration but without additional resisted sprint or ABC drill training. Prior to the intervention, all participants were engaged in regular football training consisting of routine technical, tactical, and conditioning exercises. The intervention was conducted during the in-season period.

Prior to the pre-test measurements, all participants completed two familiarization sessions to ensure proper understanding and correct execution of the testing procedures and training exercises.

Each training session was performed after a standardized warm-up and consisted of six 30-m resisted sprint repetitions and sprint-specific technical drills. All sprints were performed with resistance applied using a 60-m Exer-Genie^®^ rope system. The 60-m model was selected because it allows the generation of greater horizontal resistance compared to shorter rope systems.

The resistance intensity applied to the athletes was determined using the percentage of body weight method commonly used in resisted sprint (sled-tow) training, based on the formula (%BW / 100) × body weight [51]. The Exer-Genie^®^ resistance was set at approximately 10% of each athlete’s body weight. Since the primary aim was to improve sprint acceleration, relatively low resistance loads (~ 10% BW) were used to avoid excessive disruption of sprint mechanics [24]. All resisted sprints were performed at maximal effort.

The ABC drills were performed in a controlled, submaximal manner, focusing on sprint mechanics, coordination, posture, and rhythm rather than a fixed percentage intensity. These drills were included to enhance sprint-specific neuromuscular coordination and reinforce proper acceleration mechanics.

A passive recovery period of approximately 2–3 min was provided between sprint repetitions to minimize fatigue and maintain sprint performance quality. The selection of six sprint repetitions was based on commonly used sprint training volumes in the literature, providing sufficient neuromuscular stimulus while avoiding excessive fatigue that could negatively affect sprint mechanics.

However, the actual external resistance and velocity loss were not directly quantified, which may have resulted in inter-individual differences in training stimulus.

All training sessions were supervised by the lead researcher.

### Data collection procedure

Pre-test measurements were conducted on 10 April 2025 between 16:00 and 20:00 at the Indoor Sports Hall of Istanbul Gedik University, Faculty of Sport Sciences. The first training session was performed on 14 April 2025. After the completion of the eight-week training period, post-test measurements were taken on 10 June 2025 at the same facility and within the same time interval (16:00–20:00). Outcome assessors were blinded to the group allocations during both pre- and post-intervention tests. Throughout the study period, any adverse events or injuries were systematically monitored and recorded by the team physiotherapist.

### Data analysis

The normality of the data was examined using the Shapiro–Wilk test and skewness–kurtosis values [52, 53]. The results indicated that all variables were normally distributed within acceptable ranges for normality assumptions. Data were screened for outliers and missing values prior to analysis [53]. Homogeneity of variance was assessed using Levene’s test [54]. A priori power analysis was conducted using JASP software, indicating that the study had adequate power to detect large effect sizes, although the power was limited for detecting smaller effects. Descriptive statistics were presented as mean ± standard deviation (SD). All statistical analyses were performed using JASP software [55].

To examine differences between groups over time, a two-way mixed-design analysis of variance (ANOVA) (group × time) with repeated measures on the time factor was performed [56]. This analysis allowed the evaluation of the main effects of group (experimental vs. control), time (pre-test vs. post-test), and the group × time interaction effect. Effect sizes for ANOVA were reported as partial eta squared ($\:{\eta\:}_{p}^{2}$) [57]. When significant main or interaction effects were observed, Bonferroni-adjusted pairwise comparisons were applied to determine the source of the differences [56]. Cohen’s d was calculated for pairwise comparisons to assess the magnitude of differences [58]. The level of statistical significance was set at *p* < .05 for all analyses.

### Ethics

The study protocol was approved by the Ethics Committee of Istanbul Gedik University (Date: 26.03.2025, Decision No: 2025/4, Reference No: E-56365223-050.04-2025.137548.86). The study was conducted in accordance with the principles of the Declaration of Helsinki. The trial protocol was retrospectively registered at ClinicalTrials.gov (Identifier: NCT07274566). Furthermore, this study was conducted and reported in adherence to the Consolidated Standards of Reporting Trials (CONSORT) guidelines.

## Discussion

The present study aimed to examine the effects of an eight-week Exer-Genie^®^-assisted resisted sprint and ABC drill training program on sprint performance, hamstring muscle strength, and vertical jump performance in young female football players. The main findings indicated that the intervention group showed significant improvements in short-distance acceleration (0–10 m) and overall 30 m sprint performance compared to the control group. In addition, substantial increases were observed in isometric hamstring strength and impulse production, whereas vertical jump performance showed only a numerical improvement without a significant group × time interaction.These findings suggest that resisted sprint training primarily enhances early acceleration and hamstring function rather than maximal velocity or vertical jump performance.

Following eight weeks of Exer-Genie–assisted speed and ABC drill training, a significant improvement was observed in 30-m sprint performance. The mean 30-m sprint time of the intervention group decreased by approximately 3.8%, whereas the control group demonstrated only negligible changes. Importantly, the significant group × time interaction in favor of the experimental group indicates that the observed improvements were greater than those in the control group, suggesting a likely intervention-related effect. This finding suggests that resisted sprint training may contribute to improvements in acceleration and overall sprint performance in young female football players. Indeed, the literature reports that interventions of similar duration meaningfully improve 20–30 m sprint performance. In a 2024 study, Lee et al. reported that an eight-week SAQ (speed, agility, quickness) training program significantly improved 20-m and 30-m sprint times in elite U20 female football players (*p* < .05) [59]. Systematic reviews on resisted sprint training have also shown that 6–8 weeks of resistance-based sprint interventions provide substantial gains in short-distance acceleration. In a 2025 review conducted by Espasa et al. on rugby athletes, weighted sprint training performed at ~ 10–13% of body mass over 6–8 weeks produced significantly greater improvements in 5–20 m sprint times compared to traditional sprint training [60]. These findings are consistent with the sprint improvements observed in the present study and suggest that Exer-Genie^®^-assisted training may be a useful field-based approach for supporting sprint performance development.

Possible mechanisms underlying these improvements include increased horizontal force production and neural adaptations [61, 62]. Resisted sprint running requires athletes to apply greater horizontal force to the ground during the acceleration phase, thereby improving acceleration capacity [63]. In addition to mechanical factors, neural adaptations may have contributed to these improvements. Resisted sprint training is known to enhance motor unit recruitment, increase neural drive, and improve intermuscular coordination, particularly during the early acceleration phase. Furthermore, improvements in rate of force development (RFD) may enable athletes to produce force more rapidly, thereby enhancing acceleration performance.In resisted towing using the Exer-Genie device, the load setting directly determines horizontal resistive force, which is also associated with rope length. The measurement study by Ghigiarelli et al. showed that the Exer-Genie can increase horizontal force output in a linear manner across different loads and rope lengths (R² = 0.96–0.99) [15]. This aligns with the improvement observed in the intervention group in the early acceleration phase, reflected by the reductions in 0–10 m and 0–30 m times.

Our 0–10 m sprint results demonstrated an approximately 4% improvement in acceleration in the intervention group (*p* < .01), whereas the control group showed no change. The improvement observed in the 0–10 m sprint phase can be explained by enhanced horizontal force production and more effective force application during early acceleration. During this phase, sprint performance is primarily determined by the athlete’s ability to generate high levels of horizontal ground reaction force while maintaining an optimal forward body lean. Resisted sprint training likely increases the rate of force development (RFD) and improves neuromuscular coordination, enabling athletes to produce force more rapidly and efficiently during the initial steps of sprinting. This early acceleration gain highlights the phase-specific effect of resisted sprint running with the Exer-Genie^®^. The 10–30 m interval also improved in the intervention group (2.3% reduction, *p* < .01); however, the absence of a significant group × time interaction suggests that this improvement was not meaningfully different from the control group. Because this distance represents the transition toward maximal velocity, limited gains are expected, as achieving top-speed improvements typically requires longer-duration or plyometric-supported programs. Consistent with this, Aloui et al. observed moderate improvements in 5–30 m sprint times and significant increases in vertical jump height following an eight-week combined sprint and plyometric program among elite youth football players [64].

Our findings similarly demonstrate that combining Exer-Genie–based resisted sprint training with technical agility drills (ABC) is effective for improving short- and mid-distance sprint performance. Furthermore, Castaño-Zambudio et al. reported that high-speed running alone improved 30-m sprint performance in professional female football players during the in-season period; however, adding resisted sprint training further enhanced acceleration gains. Accordingly, it has been emphasized that a balanced integration of high-speed game-based drills and resisted running yields the best performance outcomes [65]. The current study supports this approach and indicates that a combined training model can produce meaningful sprint improvements even within a short preparatory period.

Another important finding of this study is the marked increase in hamstring muscle strength. In the intervention group, maximal prone isometric hamstring strength (averaged across right and left limbs) improved by approximately 20%, whereas the control group demonstrated increases of only ~ 7–8%. Although both groups showed within-group strength gains over time (main effect of time, *p* < .001), the significantly greater improvements in the intervention group (time × group interaction, *p* < .001) indicate that Exer-Genie^®^–assisted training is particularly effective for enhancing hamstring strength.This interaction effect suggests that the magnitude of improvement was greater in the intervention group than in the control group, supporting a likely intervention-related effect.This increase may be attributed to the continuous eccentric loading imposed on the hamstrings during resisted acceleration efforts [63, 66].

Consistent with this, the comparative study conducted by Mendiguchía et al. reported that football players performing only the Nordic Hamstring Exercise did not show improvements in sprint performance, whereas the group performing direct sprint training demonstrated significant reductions in 5-m and 20-m sprint times. Moreover, the sprint-focused training group exhibited an approximately 16% increase in the fascicle length of the biceps femoris long head, while the increase in the Nordic-only group remained limited to ~ 7%. These findings suggest that maximal sprint training induces not only strength gains but also structural adaptations in the hamstrings, whereas isolated hamstring strengthening alone may have limited transferability to complex tasks such as sprinting [67]. Therefore, resisted running performed with the Exer-Genie^®^ may provide eccentric stimuli similar to the Nordic exercise while simultaneously eliciting specific neural adaptations that directly support sprint performance [44, 63, 67].

Supporting this, Gülü and Doğan reported that a six-week Nordic hamstring program produced small-to-moderate improvements in 30-m sprint and vertical jump performance in young athletes, demonstrating that gains in eccentric hamstring strength can translate into improved sprint capacity [66]. Another study has shown that increasing hamstring muscle strength significantly reduces the incidence of hamstring injuries. In a comprehensive 2024 meta-analysis, Nunes et al. reported that the Nordic Hamstring Exercise improves both sprint performance and eccentric hamstring strength and can prevent hamstring injuries by up to 51% [68]. This finding aligns with our interpretation that the improvements in hamstring strength observed in the current study may have contributed to the sprint gains. Still, bilateral strength increases likely contributed positively to the athletes’ overall performance profiles and may be considered a protective adaptation that can help reduce potential injury risk [67, 68].

Our study demonstrated increases not only in maximal hamstring strength but also in the hamstring muscle group’s impulse-generation capacity. In the isometric hamstring tests, the intervention group showed a marked increase in impulse integral (the 0–300 ms force–time curve area) in both legs (left: +35.6%; right: +35.7%). In contrast, the control group exhibited more modest increases (~ 12–16%). Statistical analyses revealed a significant group effect for the left hamstring impulse value (*p* = .037), whereas the main group effect for the right hamstring impulse was not significant (*p* = .178). However, the significant time × group interactions for both legs (*p* < .01) indicate that Exer-Genie training effectively enhanced the hamstrings’ capacity to generate force rapidly and sustain it over time. These findings, supported by significant group × time interactions, suggest that the improvements were likely associated with the applied training stimulus.

Enhanced horizontal force production increases hamstring loading during hip extension, thereby augmenting the total propulsive impulse produced [22, 63]. Indeed, in a 2023 study, Bramah examined the relationship between sprint mechanics and hamstring function, emphasizing that increased ground-contact time and elevated horizontal braking/propulsive demands result in greater eccentric loading on the hamstrings [63]. The hamstring impulse improvements observed in the present study may reflect this mechanism, indicating that resisted running strengthens the hamstrings’ propulsive capacity as a phase-specific adaptation to early acceleration demands [44, 63].

On the other hand, no substantial effect was observed on right–left hamstring strength asymmetry. After the eight-week training period, neither group exhibited statistically meaningful changes in bilateral imbalance (interaction *p* > .10). In the intervention group, the right–left difference decreased slightly from 6.5% to 7.2% (~ 0.7% reduction), whereas in the control group the imbalance increased from 4.0% to 10.4% (~ 6% increase). However, this differentiation did not reach significance due to insufficient sample size, and the group × time interaction was non-significant. Incorporating unilateral eccentric exercises (e.g., single-leg Nordic hamstring, single-leg Romanian deadlift) into training programs may help address inter-limb differences over time [69].

In the literature, the relationship between lower-limb strength asymmetry and sprint or change-of-direction performance is generally reported as small and inconsistent [66, 69]. Fox et al.’s meta-analysis showed that strength asymmetries of around 10% may produce only very small negative effects on sprint and change-of-direction (COD) performance (*r* ≈ − .20), while having no meaningful effect on vertical jump performance [69]. Similarly, the 2024 study by D’Hondt et al. reported that, in long-distance runners, most asymmetry metrics were not significantly associated with performance, and that small asymmetries could be tolerated without functional impairment [70]. In light of these findings, the ~ 5–7% hamstring strength asymmetry observed in our study can be considered non-critical in terms of performance. (Table 1).

**Table 1:** Descriptive characteristics of the participants

Group	*n*	Age (years)	Height Pre (cm)	Height Post (cm)	Weight Pre (kg)	Weight Post (kg)
C	17	14.82 ± 1.29	160.9 ± 5.9	159.2 ± 6.2	52.21 ± 8.16	52.30 ± 8.03
E	20	14.00 ± 1.52	158.6 ± 4.5	158.1 ± 4.9	52.36 ± 7.48	52.83 ± 7.25

As presented in Table 1, no statistically significant differences were observed between the experimental (E) and control (C) groups at baseline for age, height, and body mass (*p*> .05), confirming baseline comparability between the groups. Additionally, no significant changes were identified in these anthropometric variables over the eight-week training period

Although our training program did not specifically target vertical jump enhancement, improvements in vertical jump height were observed after the eight-week period. The mean jump height in the intervention group increased by 12.4%, rising from 21.2 cm to 23.8 cm. In contrast, the control group exhibited only a modest improvement of ~ 2.5% (from 19.5 to 19.98 cm). However, no significant group × time interaction was observed (*p* = .094), indicating that the improvement in the experimental group was not statistically different from the control group.Nevertheless, although CMJ height increased numerically in the experimental group, the absence of a significant group × time interaction indicates that this finding should be interpreted cautiously and cannot be clearly attributed to the intervention. (Table 2).

**Table 2:** 30-m sprint test – pre- and post-test results

Parameter	Group	Pre-Test (Mean ± SD)	Post-Test (Mean ± SD)	F	*p*	$\:{\eta\:}_{p}^{2}$	Cohen’s d
0–10 m sprint (s)	C	2.150 ± 0.10	2.154 ± 0.12	10.17	0.003	0.225	-0.042
	E	2.102 ± 0.10	2.020 ± 0.07				0.835
10–30 m sprint (s)	C	3.232 ± 0.18	3.208 ± 0.18	0.709	0.406	0.020	0.111
	E	3.045 ± 0.29	2.974 ± 0.16				0.333
30 m sprint – best time (s)	C	5.401 ± 0.27	5.391 ± 0.28	21.08	< 0.001	0.376	0.040
	E	5.197 ± 0.25	5.002 ± 0.23				0.762

The mixed-design ANOVA revealed a significant main effect of time for all parameters (*p* < .05), indicating a general change in sprint performance over the intervention period. Most importantly, a significant group × time interaction was observed for the 0–10 m sprint [F_(1, 35)_ = 10.17, *p*=.003, $\:{\eta\:}_{p\:}^{2}$=0.225] and the 30-m sprint best time [F_(1, 35)_ = 21.08, *p*<.001,$\:{\eta\:}_{p}^{2}$=0.376]. These findings suggest that the groups responded differently to the training protocols over time.

Pairwise comparisons and effect size analyses further clarified these differences:0–10 m Sprint: The experimental group showed a significant improvement from pre-test to post-test with a large effect size (Cohen’s d = 0.835), whereas the control group remained stable (d= -0.042)

10–30 m Sprint: Although the interaction effect did not reach statistical significance [*p*= .406,$\:{\eta\:}_{p}^{2}$ = 0.020], the experimental group exhibited a small effect size (d = 0.333) compared to the insignificant change in the control group (d = 0.111)

30-m Sprint Best Time: A substantial improvement was observed in the experimental group, supported by a large effect size (d = 0.762), while the control group showed minimal variation (d = 0.040)

*C* Control Group, *E* Experimental Group, *SD* Standard Deviation, $\:{\eta\:}_{p}^{2}$ Partial Eta Squared, *Cohen’s d* Effect size for within-group comparisons

The literature likewise reports that sprint- and plyometric-based programs can positively influence both horizontal speed and vertical jump capacity. Thurlow et al.’s 2024 meta-analysis showed that repeated sprint training produces small but significant improvements in vertical jump height among athletes (effect size g ≈ 0.26) [71]. Similarly, in a 2024 study by Darragi et al., elite female football players who completed a 12-week in-season strength-training program demonstrated substantial improvements in countermovement jump (CMJ) performance compared with a control group (d = 1.27). Interestingly, no differences were found between groups in 5-m, 10-m, or 30-m sprint times. The authors emphasized that while strength training enhances general power and jumping ability, improvements in short-distance sprint performance require specific sprint or plyometric components [72]. (Table 3).

**Table 3:** ISO Prone hamstring maximum force – pre- and post-test results

Parameter	Group	Pre-Test (Mean ± SD)	Post-Test (Mean ± SD)	F	*p*	$\:{\eta\:}_{p}^{2}$	Cohen’s d
Left max (N)	C	189.0 ± 35.3	196.8 ± 31.9	15.94	< 0.001	0.313	− 0.184
	E	195.7 ± 46.2	234.3 ± 51.3				− 0.906
Right max (N)	C	197.9 ± 39.4	219.7 ± 29.0	8.189	0.007	0.190	− 0.596
	E	208.3 ± 39.5	250.1 ± 36.8				− 1.143

Table 3 illustrates the pre- and post-test results for isometric (ISO) prone hamstring maximum force across both left and right limbs for the experimental (E) and control (C) groups. Statistical analysis via mixed-design ANOVA revealed significant group × time interaction effects for both the left maximum force [F_(1, 35)_ = 15.94, *p* < .001,$\:{\eta\:}_{p\:}^{2}$= 0.313] and the right maximum force [F_(1, 35)_ = 8.189, *p* = .007,$\:{\eta\:}_{p\:}^{2}$= 0.190]. These results indicate that the changes in hamstring strength over the intervention period differed significantly between the two groups, with the experimental group demonstrating superior gains

Further examination of within-group changes and effect sizes provided deeper insights: Left Hamstring Max Force: The experimental group exhibited a substantial increase in force production, moving from 195.7 ± 46.2 N to 234.3 ± 51.3 N, representing a large effect size (Cohen’s d= -0.906). In contrast, the control group showed a negligible increase with a small effect size (d= -0.184). Right Hamstring Max Force: Similar to the left limb, the experimental group achieved a significant improvement (208.3 ± 39.5 N to 250.1 ± 36.8 N), characterized by a very large effect size (d= -1.143). While the control group also showed some improvement (d= -0.596), the magnitude of change was markedly lower than that of the experimental group

*C* Control Group, *E* Experimental Group, *SD* Standard Deviation,$\:{\eta\:}_{p}^{2}$Partial Eta Squared, *Cohen’s d* Effect size for within-group comparisons

Our findings align with this perspective: vertical jump performance remained nearly unchanged in the control group, whereas the combined sprint-plus-technical training implemented in the intervention group produced a greater numerical improvement. Potential explanations for this increase include adaptations in lower-limb strength and neuromuscular performance capacities [71, 72]. Resisted sprints likely strengthened the hamstring and gluteal muscles through repeated eccentric–concentric loading cycles, while ABC drills may have enhanced coordination and movement efficiency, thereby contributing to more effective force production. (Table 4).

**Table 4:** ISO Prone hamstring mean force – pre- and post-test results

Parameter	Group	Pre-Test (Mean ± SD)	Post-Test (Mean ± SD)	F	*p*	$\:{\eta\:}_{p}^{2}$	Cohen’s d
Left average (N)	C	184.2 ± 34.0	186.5 ± 26.3	10.83	0.002	0.236	− 0.060
	E	189.6 ± 42.9	217.8 ± 43.9				− 0.741
Right average (N)	C	191.9 ± 35.6	207.9 ± 28.7	5.502	0.025	0.136	− 0.461
	E	202.6 ± 37.4	233.7 ± 35.3				− 0.899

Table 4 presents the pre- and post-test mean isometric force results for the prone hamstring across the left and right limbs. The mixed-design ANOVA indicated significant group × time interaction effects for both the left average force [F_(1, 35)_ = 10.83, *p*= .002, $\:{\eta\:}_{p\:}^{2}$= 0.236] and the right average force [F_(1, 35)_ = 5.502, *p*= .025, $\:{\eta\:}_{p\:}^{2}$= 0.136]. These findings demonstrate that the intervention led to significantly different patterns of strength development between the experimental (E) and control (C) groups over the course of the study

Specific within-group changes and their corresponding effect sizes revealed the following: Left Hamstring Mean Force: The experimental group showed a significant increase in mean force production (189.6 ± 42.9 N to 217.8 ± 43.9 N), which is associated with a moderate-to-large effect size (Cohen’s d= -0.741). Conversely, the control group exhibited a negligible change (d= -0.060), indicating no substantial improvement in the left limb

Right Hamstring Mean Force: Both groups showed improvements in the right limb; however, the magnitude of change was considerably higher in the experimental group (202.6 ± 37.4 N to 233.7 ± 35.3 N) with a large effect size (Cohen’s d= -0.899). The control group demonstrated a more modest improvement (d= -0.461), which represents a medium effect size

*C* Control Group, *E* Experimental Group, *SD* Standard Deviation, $\:{\eta\:}_{p}^{2}$ Partial Eta Squared, *Cohen’s d* Effect size for within-group comparisons

The literature shows that combined plyometric and sprint training improves both horizontal and vertical jump performance in young athletes [64, 73]. In the eight-week study by Aloui et al., plyometric and short-distance sprint training produced notable gains in squat and jump tests among elite U17 football players [64]. Consistent with this, the Exer-Genie resisted running used in our study may have created a form of horizontal plyometric stimulus, contributing to increased explosive power capacity in the athletes. Additionally, the hamstring-focused eccentric loading inherent in our program may have indirectly contributed to improvements in jumping mechanics. Potosí-Moya et al. conducted a randomized controlled trial in multi-sport young athletes in which a seven-week Nordic hamstring exercise program was applied. They reported that hamstring strength increased significantly in both legs in the intervention group and that vertical jump height improved by approximately + 3.4 cm on average. Similarly, in the present study, the intervention group exhibited a 12.4% increase in jump height; however, this improvement should be interpreted with caution given the non-significant interaction effect (*p* = .094) [74]. (Table 5).

**Table 5:** ISO Prone hamstring impulse – pre- and post-test results

Parameter	Group	Pre-Test (Mean ± SD)	Post-Test (Mean ± SD)	F	*p*	$\:{\eta\:}_{p}^{2}$	Cohen’s d
Left impulse (N·s)	C	1782 ± 330	1995 ± 413	10.62	0.002	0.233	− 0.481
	E	1841 ± 405	2496 ± 569				− 1.478
Right impulse (N·s)	C	2017 ± 482	2339 ± 424	9.753	0.004	0.218	− 0.667
	E	2019 ± 523	2739 ± 490				− 1.491

Table 5 presents the pre- and post-test results for isometric prone hamstring impulse for both the left and right limbs. The mixed-design ANOVA revealed highly significant group × time interaction effects for the left impulse [F_(1, 35)_ = 10.62, *p*= .002, $\:{\eta\:}_{p\:}^{2}$= 0.233] and the right impulse [F_(1, 35)_ = 9.753, *p*= .004, $\:{\eta\:}_{p\:}^{2}$= 0.218]. These results indicate that the intervention protocol led to significantly different rates of improvement in impulse production between the experimental (E) and control (C) groups over the measurement period

Detailed within-group analyses and effect size calculations further support the efficacy of the intervention: Left Hamstring Impulse: The experimental group demonstrated a substantial increase in impulse from 1841 ± 405 N·s to 2496 ± 569 N·s, characterized by a very large effect size (Cohen’s d= -1.478). In comparison, the control group showed a more modest increase (d= -0.481), representing a small-to-medium effect size.

Right Hamstring Impulse: Similarly, the experimental group achieved a marked improvement in the right limb (2019 ± 523 N·s to 2739 ± 490 N·s), yielding a very large effect size (d= -1.491). While the control group also exhibited an increase (d= -0.667), the magnitude of this change was considerably smaller than that observed in the experimental group

*C* Control Group, *E* Experimental Group, *SD* Standard Deviation, $\:{\eta\:}_{p}^{2}$ Partial Eta Squared, *Cohen’s d* Effect size for within-group comparisons

The authors emphasized that eccentric training targeting the hamstring muscles can improve lower-limb force production and jumping ability, and that increased muscle length and endurance may also help reduce injury risk [75]. Overall, our findings highlight the multifaceted relationship among sprint performance, hamstring strength, and vertical jump capability. Exer-Genie^®^-assisted resisted sprint training not only improved short-distance acceleration ability in young female football players but also increased the force-production capacity of the lower-limb muscles. (Table 6).

**Table 6:** Countermovement jump performance

Group	Pre-Test (cm)	Post-Test (cm)	F	*p*	$\:{\eta\:}_{p}^{2}$	Cohen’s d
C	19.50 ± 3.76	19.98 ± 3.33	2.957	0.094	0.078	− 0.111
E	21.18 ± 4.83	23.81 ± 4.78				− 0.614

Table 6 presents the pre- and post-test results for Countermovement Jump (CMJ) height for the experimental (E) and control (C) groups. The mixed-design ANOVA indicated that the group × time interaction effect did not reach the threshold for statistical significance [F_(1, 35)_ = 2.957, *p*= .094, $\:{\eta\:}_{p\:}^{2}$= 0.078]. However, the partial eta squared value ($\:{\eta\:}_{p\:}^{2}$= 0.078) suggests a medium effect size for the interaction, indicating a trend toward different response patterns between the two groups over time

A closer examination of within-group changes and Cohen’s d effect sizes provides further context for these results: Experimental Group (E): The experimental group demonstrated a noticeable improvement in jump height, increasing from 21.18 ± 4.83 cm to 23.81 ± 4.78 cm. This change is associated with a moderate effect size (Cohen’s d= -0.614), suggesting a practical enhancement in explosive power following the intervention.

Control Group (C): In contrast, the control group showed minimal variation in performance (19.50 ± 3.76 cm to 19.98 ± 3.33 cm), which corresponds to a trivial effect size (Cohen’s d= -0.111)

*C* Control Group, *E* Experimental Group, *SD* Standard Deviation, $\:{\eta\:}_{p}^{2}$ Partial Eta Squared, *Cohen’s d* Effect size for within-group comparisons

As emphasized in the literature, enriching sprint training with appropriate modalities and tools (e.g., weighed running, plyometric exercises) can simultaneously enhance explosive performance in both the horizontal and vertical planes. Our study indicates that integrating portable resistance devices such as the Exer-Genie^®^ with traditional speed and technical training is an effective and practical approach for improving field performance, particularly in female athletes aged 13–17 years.

### Limitations

The present study has some limitations. First, the sample size was relatively small, although satisfied by power analysis. Second, the duration of the intervention was limited to eight weeks; longer periods might yield different physiological adaptations. Finally, nutritional intake and daily physical activity levels outside of training were not strictly monitored. In addition, the external resistance applied using the Exer-Genie device was prescribed based on body mass rather than individualized velocity loss, which may have resulted in variability in the training stimulus between participants. Furthermore, the training content of the control group was not strictly standardized, which may have influenced the comparison between groups.

## Conclusion

This study demonstrated that eight weeks of Exer-Genie^®^-assisted speed and ABC drill training significantly improved short-distance acceleration (0–10 m) and overall sprint performance (30 m) in young female football players, with significant group × time interactions suggesting that these improvements were associated with the intervention. In parallel, substantial increases in hamstring strength (~ 20%) and impulse production (~ 35%) indicate enhanced force-generating capacity, particularly during the early acceleration phase. No significant changes were observed in hamstring strength asymmetry, suggesting that performance gains were achieved without inducing imbalances. Although vertical jump height increased numerically, the absence of a significant interaction effect indicates that this outcome should be interpreted with caution. Overall, these findings suggest that Exer-Genie^®^-assisted resisted sprint training may be a practical field-based method for improving acceleration performance and hamstring function in youth female football players.

## Supplementary Information

Supplementary Material 1.

## Data Availability

The datasets generated and analyzed during the current study are available from the corresponding author on reasonable request.
